# Hospitalization and ambulatory care in imported-malaria: evaluation of trends and impact on mortality. A prospective multicentric 14-year observational study

**DOI:** 10.1186/s12936-016-1364-9

**Published:** 2016-06-07

**Authors:** Enrique Casalino, Aurélie Etienne, France Mentré, Sandrine Houzé, The Imported Malaria Hospitalization Study Group

**Affiliations:** Service d’Accueil des Urgences, Assistance Publique-Hôpitaux de Paris (AP-HP), Hôpital Bichat-Claude Bernard, 46 rue Henri Huchard, 75018 Paris, France; Université Paris Diderot, PRES Sorbonne Paris Cité, EA 7334 «Recherche clinique coordonnée ville-hôpital, Méthodologies et Société (REMES)», Paris, France; INSERM, IAME, UMR 1137, 75018 Paris, France; Service de Biostatistique, AP-HP, Hôpital Bichat, 75018 Paris, France; Université Paris Diderot, Sorbonne Paris Cité, 75018 Paris, France; Parasitology Laboratory, Centre National de Référence du Paludisme, Assistance Publique-Hôpitaux de Paris (AP-HP), University Hospital Bichat-Claude Bernard, Paris, France; RD UMR216, Mère et enfant face aux infections tropicales, 75006 Paris, France; Faculté des Sciences Pharmaceutiques et Biologiques, PRES Sorbonne Paris Cité, Université Paris Descartes, 75270 Paris, France; Centre National de Référence du Paludisme, Hôpital Bichat, 46 rue Henri Huchard, 75018 Paris, France

**Keywords:** Imported malaria, Hospitalization, Ambulatory care, Intensive care, Mortality

## Abstract

**Background:**

Hospitalization is usually recommended for imported malaria. The goal of the present study is to evaluate the evolution in clinical pathways while measuring their impact on mortality.

**Methods:**

This is a 14-year prospective observational study divided into three periods. We evaluated for adult (≥15 years) and paediatric (<15 years) case trends in severity, clinical pathways (hospitalization in medical ward (MW) or intensive care unit (ICU), ambulatory care) and mortality.

**Results:**

In total, 21,386 imported malaria cases were included, 4269 of them were paediatrics (20 %). Rises in severe forms for adults [from 8 % in period 1–14 % in period 3 (p = 0.0001)] and paediatrics [from 12 to 18 % (p < 0.0001)] were found. For adults, MW admission rates decreased [−15 % (CI 95 % −17; −13)] while ambulatory care [+7 % (CI 95 % 5–9)] and ICU admission rates [+4 % (CI 95 % 3–5)] increased. For paediatrics, increase in ICU admissions (+3 %) was shown. We did not observe any change in overall mortality during the study periods, whether among adults or children, regardless of care pathway.

**Conclusions:**

The present study indicates a changing management of imported malaria in adults, with an increasing trend for ambulatory care. The absence of change in mortality for adults indicates that ambulatory care can be proposed for adults presenting non-severe imported malaria.

**Electronic supplementary material:**

The online version of this article (doi:10.1186/s12936-016-1364-9) contains supplementary material, which is available to authorized users.

## Background

Malaria is diagnosed in 19–29 % of symptomatic travellers, mainly *Plasmodium falciparum* malaria [1, 2]. There are 1500 cases in the USA and 12,000 cases per year in Europe, of which 4000 are in France [3–8]. Paediatric cases represent 15–20 % of imported malaria cases [9]. Patient management and access to care is dependent on clinical or biological severity [10–12]. Most guidelines call for systematic hospitalization for imported falciparum malaria [12–15]. In 1999, French guidelines recommended systematic short hospitalization for falciparum malaria [8], and in 2007 ambulatory care for non-severe forms of *P. falciparum* imported malaria in adults [16]. Hospitalization rates for imported malaria vary between 30 and 72 % [3, 17, 18]. All guidelines recommend that severe falciparum malaria be managed in intensive care units (ICU) [8, 12–16]. However, ICU admission frequency varies between 2.6 and 5 % for adults [17, 18] against 7.4 % for children [9, 19].

The hypothesis of this work was that revision of guidelines, the availability of new oral treatments and the evolution of patients’ characteristics could have modified clinical practices in malaria case management in recent years. The aim was to identify trends in the management of malaria cases in Ile-de-France between 2000 and 2013. Over the study period, hospitalization rates in general medical ward (MW), intensive care units (ICU) and the proportion of patients in ambulatory care were identified, as well as their impact on mortality according to care pathway.

## Methods

### Study design and data source

In this observational study, data was collected prospectively by the French National Reference Centre for Malaria (CNR-M) in metropolitan France, a network of about 100 hospitals. For each confirmed malaria case (positive blood smear), physicians in the network’s hospitals complete a standard case report form (CRF) with demographic, epidemiological, clinical, and parasitological data.

### Study population

The study population consisted of all malaria cases reported by hospitals of the CNR located in Ile-de-France (Paris area) in order to ensure a greater degree of homogeneity in terms of population, access to health care and case management. First, the population is more homogeneous in this region of 12 million inhabitants, with a high proportion of African migrants. Moreover, 45 % of the 44 hospitals are academic hospitals, 25 % have infectious disease units, and others are major hospitals with expertise in the management of malaria cases. Finally, a large proportion of imported malaria cases are reported by the hospitals located in Ile-de-France (54 % of imported malaria cases in 2013, CNR database, data not published). Data were collected prospectively and in a consistent manner, regardless of severity or type of care pathway. Children under 15 years of age were considered as paediatric cases.

### Study period

Cases reported between January 2000 and December 2013 were included. The study period was divided into three periods: Period 1: 2000–2003, Period 2: 2004–2008 and Period 3: 2009–2013. The first guidelines for the management of imported malaria cases were put forward by the French Infectious Diseases Society in 1999 [8]. These recommendations were revised in 2007 and published in 2008 [16]. Then, first and third study periods started in 2000 and 2009, respectively, i.e., 1 year after the publication of each recommendation. Since 2003, new therapeutic oral administration options have been used as first-line treatment for imported malaria. The second period started in 2004.

### Ethics statement

Data collection and storage by the CNR-M was approved by the French National Commission for Data Protection and Liberties (CNIL). Anonymized data have been extracted from the CNR-M database. The Ethics Committee for Biomedical Research of Paris-Nord approved this study.

### Study endpoint definitions

#### Severe cases

Two different definitions were used to classify malaria cases as severe cases. First, the clinical classification of the CNR-M: physicians rated patients according to their clinical judgment including patients clinical evaluation and social context as: (i) asymptomatic malaria; (ii) uncomplicated malaria without vomiting; (iii) uncomplicated malaria with vomiting; (iv) severe malaria; or, (v) evolving visceral malaria. Second, the French criteria for severe falciparum malaria [16] that were adapted to imported malaria and its management in a European context. These criteria were used for the entire study period, even if the recommendations were published in 2008 (see Additional file 1). Severity was defined by the presence of at least one severity criterion.

#### Type of care pathways

Patients were considered as admitted to the ICU or MW if they had spent at least 1 day in ICU or in MW. Length of stay (LOS) in the MW and ICU was recorded.

#### Mortality

All deaths attributable to malaria were reported to the CNR by hospital correspondents and in the same way for hospitalized and ambulatory patients. In France, all deaths and their causes are reported to the *Centre d’épidémiologie sur les causes médicales de décès* [20]. This entity informs the CNR of any certificate indicating malaria as the leading or contributing cause of death.

#### Statistical analysis

Data were aggregated by month for assessment of trends. Trends in the monthly number of cases, severe cases and in the different pathways (ambulatory care or hospitalization in MW or ICU) were displayed using time series analysis and smoothed with a two-degree polynomial regression. The trend in the number of malaria cases over the study period was tested using a simple linear regression. To describe the study population, quantitative variables were described with mean and standard deviation, and qualitative variables with numbers of patients and percentages. Chi 2 or Fisher test and Student or Wilcoxon tests were used to compare qualitative and quantitative variables between the three study periods. Description and tests were performed for adult and paediatric cases together and separately for main outcomes (number of cases, proportion of severe cases and ambulatory care or hospitalization rates) and separately for MW and ICU admissions. The significance threshold was 0.05. Missing data were not discarded. Statistical analyses were performed using R 3.1.2 software.

## Results

Between 2000 and 2013, 21,386 malaria cases were reported to the CNR (see details in Additional file 2; Additional file 3 presents a flow chart of the study population). The total number of cases decreased during the study period (see Additional file 4). The trend line for all cases decreased significantly by 99 cases per year (p < 10^−4^). The decrease was significant both in adult cases (63 cases per year, p < 10^−4^) and in paediatric cases (33 cases per year, p < 10^−5^).

Table 1 presents the evolution of patients’ main characteristics over the study period. Falciparum malaria caused 88 % of all cases over the study period and this proportion increased significantly from 87 % in Period 1 to 90 % in Period 3. Paediatric cases accounted for 20 % (4269/21,386) of all cases, and there was a significant decrease during the study period (32 % in Period 1 against 18 % in Period 3). There was also a significant increase in the proportion of patients aged over 50 years.

**Table 1:** Main characteristics of adult and paediatric cases during the three study periods (Period 1: 2000–2003, Period 2: 2004–2008, Period 3: 2009–2013)

	Adults						Paediatrics					
	Period 1	Period 2	Period 3	p	Period 2 vs Period 1	Period 3 vs Period 1	Period 1	Period 2	Period 3	p	Period 2 vs Period 1	Period 3 vs Period 1
	n = 6482	n = 5514	n = 4989		Difference	Difference	n = 2066	n = 1308	n = 895		Difference	Difference
	n (%)	n (%)	n (%)		(CI 95 %)	(CI 95 %)	n (%)	n (%)	n (%)		(CI 95 %)	(CI 95 %)
Demographic characteristics												
Age (years) (mean ± SD)	36.6 (12.5)	38.3 (13.0)	40.2 (13.5)	<10^−15^	1.7 (1.3; 2.2)	3.6 (3.2; 4.2)	7.7 (4.2)	7.3 (4.2)	7.8 (4.1)	0.006	−0.4 (−0.07; −0.09)	0.1 (−0.5; 0.2)
Age categories (years)				<10^−15^						0.02		
<1							56 (3)	58 (4)	36 (4)		1 (0; 3)	1 (0; 3)
≥1–5							594 (29)	409 (31)	230 (26)		2 (−1; 6)	−3 (−7; 0)
≥5–15							1416 (69)	841 (64)	629 (70)		−5 (−7; −1)	1 (−2; 5)
≥15–30	2204 (34)	1611 (29)	1244 (25)		−5 (−7; −3)	−9 (−11; −7)						
≥30–50	3253 (50)	2817 (51)	2545 (51)		−1 (−3; 1)	−1 (−3; 1)						
≥50	1025 (16)	1086 (20)	1200 (24)		4 (3; 5)	8 (7; 10)						
Sex				0.68						0.05		
Male	4039 (62)	3493 (64)	3076 (62)		2 (−1; 3)	0 (−2; 1)	1115 (54)	717 (55)	534 (60)		1 (−2; 5)	6 (−2; 10)
Chemoprophylaxis	123	2122 (33)	1817 (33)	1094 (22)	<10^−11^	0 (−2;2)	−11 (−13;−9)	42	1184 (58)	709 (55)	436 (49)	<10^−3^
Parasitological characteristics												
*P. falciparum*	5616 (87)	4854 (88)	4494 (90)	<10^−5^	1 (0; 3)	3 (2; 5)	1831 (89)	1189 (91)	795 (89)	0.13	2 (0; 4)	0 (−2; 3)
Parasitaemia >4 %	384 (6)	371 (7)	450 (9)	<10^−5^	1 (0; 2)	3 (2; 4)	251 (12)	174 (13)	133 (15)	0.08	1 (−1; 3)	3 (0; 5)
Clinical and biological characteristics												
Impaired consciousness	66 (1)	100 (2)	166 (3)	<10^−12^	1 (0; 1)	2 (2; 3)	0 (0)	13 (1)	24 (3)	<10^−11^	1 (1; 2)	3 (2; 3)
Glasgow coma scale <11	16 (0.2)	10 (0.2)	24 (0.5)	0.36	0 (−0.3; 0.1)	0.3 (0; 0.5)	0 (0)	4 (0.3)	5 (1)	0.002	0.3 (0; 0.7)	1 (0.; 1)
Respiratory failure	26 (0.4)	20 (0.4)	15 (0.3)	0.48	0 (−0.3; 0.2)	−0.1 (−0.3; 0.1)	0 (0)	0 (0)	0 (0)	–	–	–
Cardiovascular failure	5 (0.08)	16 (0.3)	42 (1)	<10^−7^	0.2 (0.1; 0.4)	0.9 (0.05; 1)	0 (0)	3 (0.2)	6 (1)	0.0008	0.2 (−0.1; 0.5)	1 (0; 1)
Repeated seizures ≥2/24 h	4 (0.06)	4 (0.07)	10 (0.2)	0.15	0 (−0.1; 0.1)	0.1 (0; 0.3)	0 (0)	5 (0.4)	3 (0.3)	0.001	0.4 (0; 0.8)	0.3 (−0.1; 0.7)
Haemorrhage	10 (0.2)	7 (0.1)	8 (0.2)	0.85	−0.1 (−0.2; 0.1)	0 (−0.2; 0.2)	0 (0)	0 (0)	3 (0.3)	0.05	–	0.3 (−0.1; 0.7)
Jaundice	56 (1)	70 (1)	138 (3)	<10^−8^	0 (0; 1)	2 (1; 2)	0 (0)	3 (0.2)	24 (3)	<10^−8^	0.2 (−0.1; 0.5)	3 (2; 3)
Bilirubinaemia >50 µmol/L	38 (1)	66 (1)	156 (3)	<10^−15^	0 (0; 1)	2 (2; 3)	0 (0)	3 (0.2)	17 (2)	<10^−6^	0.2 (−0.1; 0.5)	2 (1; 3)
Macroscopic haemoglobinuria	5 (0.08)	12 (0.2)	35 (1)	<10^−5^	0.1 (0; .0.3)	0.1 (0; 0.1)	0 (0)	0 (0)	6 (1)	0.005	–	1 (0; 1)
Haemoglobin <7 g/dL and haematocrit <20 %	18 (0.3)	23 (0.4)	30 (0.6)	0.02	0.1 (−0.1; 0.4)	0.3 (0.1; 0.6)	0 (0)	8 (1)	9 (1)	<10^−5^	1 (0; 1)	1 (0; 2)
Hypoglycaemia(<2.2 mmol/L)	0 (0)	2 (0.04)	4 (0.08)	0.02	0.04 (0.04; 0.1)	0.08 (0.01; 0.2)	0 (0)	1 (0.08)	1 (0.1)	0.10	0.08 (−0.2; 0.3)	0.1 (−0.3; 0.4)
Acidaemia (pH < 7.25)	0 (0)	1 (0.02)	7 (0.1)	0.006	0.02 (0.05; 0.09)	0.1 (0.04; 0.3)	0 (0)	0 (0)	0 (0)	–	–	–
Hyperlactataemia (>5 mmol/L)	0 (0)	6 (0.1)	33 (1)	<10^−9^	0.1 (0; 0.2)	1 (0; 1)	0 (0)	0 (0)	2 (0.2)	0.10	–	0.2 (−0.2; 0.5)
Renal failure (creatinaemia >265 µmol/L)	59 (1)	61 (1)	90 (2)	0.002	0 (0; 0.1)	1 (0; 1)	0 (0)	4 (0.3)	2 (0.2)	0.005	0.3 (0; 0.7)	0.2 (−0.2; 0.5)
Severity												
Clinical classification	173 (3)	236 (4)	462 (9)	<10^−15^	1 (1; 2)	6 (6; 7)	45 (2)	53 (4)	61 (7)	<10^−6^	2 (1; 3)	5 (3; 6)
French criteria	451 (7)	463 (8)	649 (13)	<10^−15^	1 (0; 2)	6 (5; 7)	251 (12)	190 (15)	161 (18)	0.0001	3 (0; 5)	6 (3; 8)

Data are number (%) or mean (SD) except for the differences between Periods 2 and 1 and between Periods 3 and 1—difference (CI 95 %)

Additional file 5 indicates the use of new oral anti-malarial drugs (atovaquone-proguanil, artemether-lumefantrine, dihydroartemisinin-piperaquine) in first intention in adult cases as the first-choice treatment during Periods 2 and 3, as they were not available during period 1.

### Frequency of severe forms

According to clinical classification, 5 % (CI 95 % 4.7–5.3) of all cases (1031/21,386) were severe, and this proportion rose significantly from 3 % in Period 1 to 9 % in Period 3 (p < 10^−15^). The increase was observed both in adults (from 3 to 9 %, p < 10^−15^) and in children (from 2 to 7 %, p < 10^−6^) (Table 1). In accordance with French criteria, 10 % (CI 95 % 9.8–10.6) of all cases (2172/21,386) were severe cases and this proportion increased significantly, from 8 % in Period 1 to 14 % in Period 3 (p < 10^−15^). This increase was observed both in adults (from 7 to 13 %, p < 10^−15^) and in children (from 12 to 18 %, p = 0.0001) (Table 1 and Additional file 6).

### Frequency of clinical pathways

A total of 11,208 patients [59 % (CI 95 % 58.2–59.7)] out of the 19,012 with non-missing data for type of care were hospitalized. Over the study period, this proportion dropped significantly for adults, from 57 % in Period 1 to 50 % in Period 3 (p < 10^−10^), but not for children (from 78 to 76 %, p = 0.2) (Table 2).

**Table 2:** Clinical pathways for adult and paediatric cases during the study periods (Period 1: 2000–2003, Period 2: 2004–2008, Period 3: 2009–2013)

	Period 1	Period 2	Period 3	p value	Period 2 vs Period 1	Period 3 vs Period 1
	n (%)	n (%)	n (%)		Difference (CI 95 %)	Difference (CI 95 %)
Adult^a^	n = 5185	n = 5159	n = 4643			
Overall hospitalization	2971 (57)	2729 (53)	2335 (50)	<10^−10^	−4 (−6; −2)	−7 (−9; −5)
Medicine ward	2230 (48)	1578 (38)	1318 (34)	<10^−15^	−10 (−13; −9)	−15 (−17; −13)
MW length of stay (mean ± SD) (days)	3.9 (4.0)	4.1 (4.5)	4.2 (3.6)	0.08	0.2 (−0.4; 0.09)	0.3 (0.05; 0.5)
ICU	174 (4)	181 (4)	303 (8)	<10^−7^	0 (0; 1)	4 (3; 5)
ICU length of stay (mean ± SD) (days)	5.7 (7.1)	5.3 (8.3)	3.3 (3.5)	<10^−4^	−0.4 (−1.2; 2.0)	−2.4 (−3.3; −1.4)
Proportion of ICU/total admissions (%)	7.2	10.3	18.7	<10^−15^	3.1 (2.8; 3.4)	11.5 (11; 12)
Ambulatory care	2214 (43)	2430 (47)	2308 (50)	4.4.10^−11^	4 (2; 6)	7 (5; 9)
Children^a^	n = 1891	n = 1189	n = 847			
Overall hospitalization	1481 (78)	988 (83)	641 (76)	0.20	5 (2; 8)	−2 (−6; 1)
Medicine ward	1185 (74)	676 (74)	477 (67)	0.06	0 (−3; 4)	−7 (−11; −3)
MW length of stay (mean ± SD) (days)	2.7 (2.0)	2.5 (1.9)	2.6 (2.0)	0.08	−0.2 (−0.4; −0.03)	−0.1 (−0.3; 0.12)
ICU	14 (1)	40 (4)	30 (4)	<10^−9^	3 (2; 5)	3 (2; 5)
ICU length of stay (mean ± SD) (days)	2.5 (1.5)	2.1 (1.6)	4.6 (7.9)	0.10	−0.4(−1.4; 0.5)	2.1 (−2.2; 6.4)
Proportion of ICU/total admissions (%)	1.2	5.7	5.9	<10^−8^	4.5 (3.9; 5.2)	4.7 (4.0; 5.4)
Ambulatory care	410 (22)	201 (17)	206 (24)	0.20	−5 (−8; −2)	2 (−1; 6)

^a^There were 2251 and 688 missing values for adult and paediatric cases, respectively

**Fig. 1:**
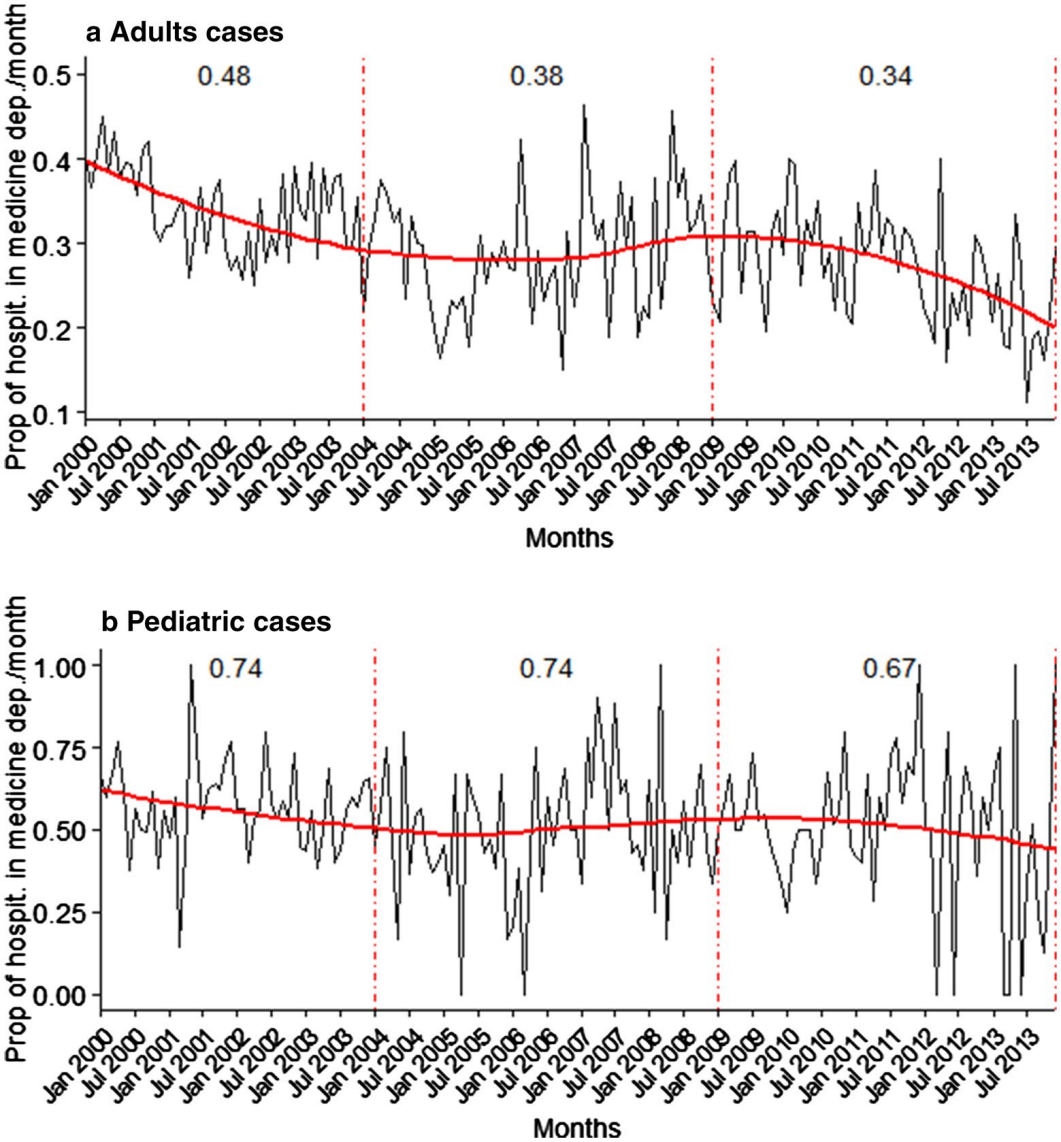
Trends in the proportion of hospitalization in medical ward during the study period among malaria cases reported in Ile-de-France, 2000–20,013, by month, according to age group. The number reported above the curve is the observed proportion for each period (Period 1: 2000–2003, Period 2: 2004–2008, Period 3: 2009–2013). The figure shows the proportion of patients with malaria cases hospitalized in the general Medical Ward, by month, among cases reported to the CNR between January 1st, 2000 and December 31, 2013 (*black lines*), smoothed with a 2^°^ polynomial regression line (*red lines*), in adults (**a**) and pediatric cases (**b**). *Dotted red lines* represent limits between the three study periods. Proportions are specified for each period

A total of 7503 patients [47 % (CI 95 % 46.0–47.5)] were hospitalized in a MW. The proportion of hospitalization in MW decreased significantly over the study period, from 55 % in Period 1 to 39 % in Period 3 (p < 10^−15^). Among adults, this proportion fell considerably, from 48 % in Period 1 to 34 % in Period 3 (p < 10^−15^) while the mean duration of stay remained globally unchanged (from 3.9 days in Period 1 to 4.2 days in Period 3, p = 0.08). However, in paediatric cases this trend was not significant (decrease from 74 % in Period 1 to 67 % in Period 3, p = 0.06), nor was the change in the mean duration of stay (from 2.7 days in Period 1 to 2.6 days in Period 3, p = 0.08) (Table 2; Fig. 1).

**Fig. 2:**
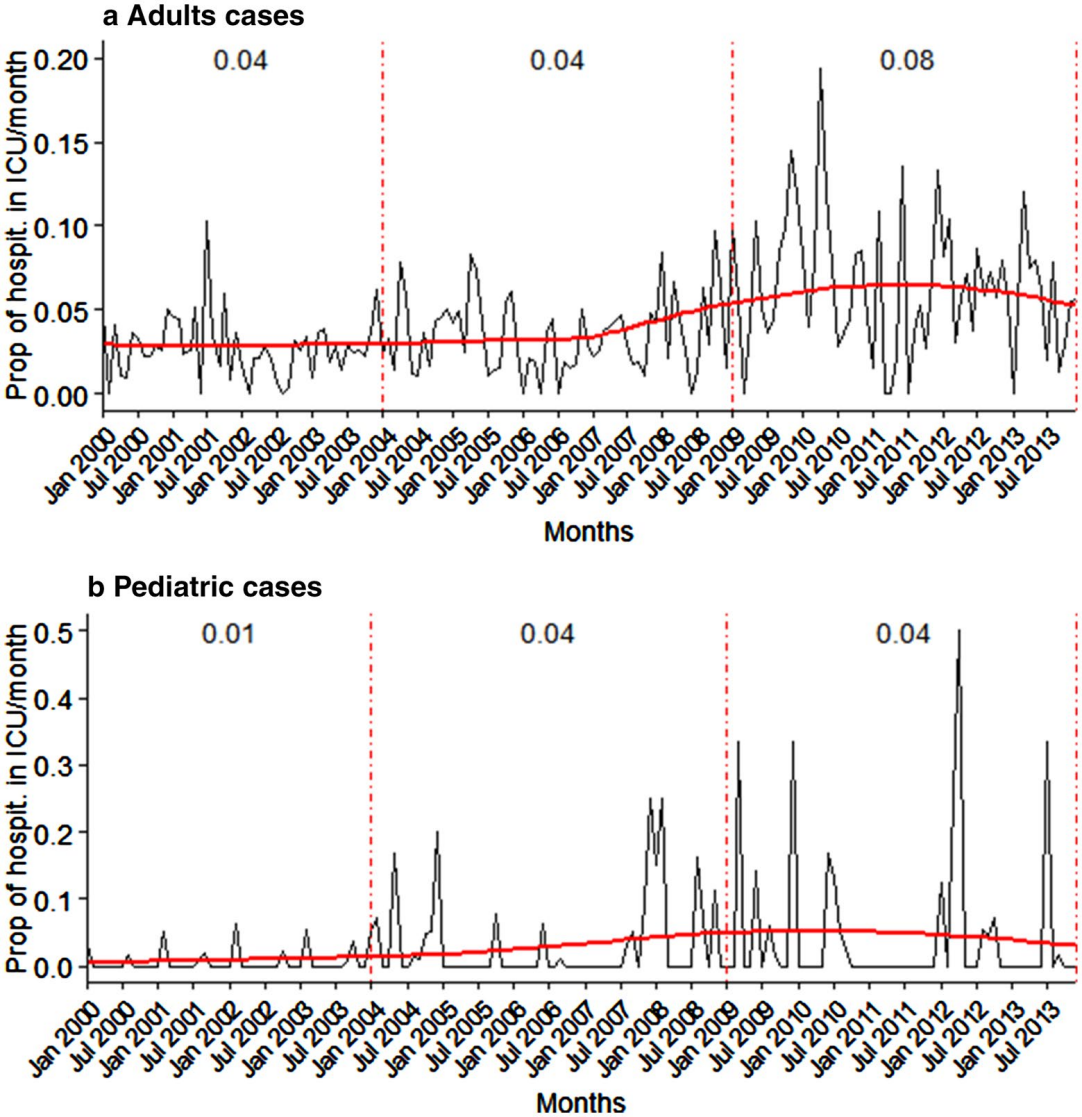
Trends in the proportion of hospitalization in intensive care units during the study period among malaria cases reported in Ile-de-France, 2000–20,013, by month, according to age group. The number reported above the curve is the observed proportion for each period (Period 1: 2000–2003, Period 2: 2004–2008, Period 3: 2009–2013). The figure shows the proportion of patients with malaria cases hospitalized in ICU, by month, among cases reported to the CNR between January 1st, 2000 and December 31, 2013 (*black lines*), smoothed with a 2^°^ polynomial regression line (*red lines*), in adults (**a**) and pediatric cases (**b**). *Dotted red lines* represent limits between the three study periods. Proportions are specified for each period

A total of 742 patients [4.3 % (CI 95 % 4. 6–5.0)] were admitted to an ICU. The proportion of ICU hospitalizations rose significantly over the study period from 3 % in Period 1 to 7 % in Period 3 (p < 10^−15^). Among adults, this proportion increased from 4 % in Period 1 to 8 % in Period 3 (p < 10^−7^). Conversely, the mean duration of stay in ICU fell from 5.7 days to 3.3 days (p < 10^−4^). In paediatric cases, this increase in ICU hospitalization was also significant, rising from 1 % in Period 1 to 4 % in Period 3 (p < 10^−9^), while the mean duration of stay in ICU did not change significantly (from 2.5 days in Period 1 to 4.6 days in Period 3, p = 0.1) (Table 2; Fig. 2).

The proportion of patients admitted to an ICU compared to all admitted patients (ICU/ICU + MW) was calculated. As presented in Table 2, there is an increasing proportion of ICU admission between Period 1 and Period 3 among adults [from seven in Period 1 to 19 in Period 3 (p < 10^−15^)] and children [from 1.2 to 6 (<10^−8^)].

Eighty-eight per cent of adults [1261/1427 (CI 95 % 87–90)] and 89 % (CI 95 % 86–91) of children (504/567) with severe malaria according to French criteria were hospitalized, whereas this proportion rose to 98 % of adults (801/816) and 99 % of children (151/152) classified as severe malaria cases by physicians. Among hospitalized adults with severe malaria according to French criteria, 49 % (452/920) were admitted to an ICU, but this proportion rose to 72 % (422/590) for those classified as severe cases by physicians. Among paediatric patients hospitalized, 15 % (54/372) of severe cases according to French criteria and 51 % (52/101) of severe cases according to clinical classification were admitted to an ICU.

### Mortality

Of the 21,386 reported cases, 57 died: 54 adults and three children [overall mortality: 0.27 % (CI 95 % 0.2–0.35); adults 0.32 % (CI 95 % 0.24–0.41) and paediatrics 0.07 % (CI 95 % 0.01–0.21)]. Adult and paediatric mortality rates, in ICU were 5.47 and 2.38 %, respectively, whereas five adults died in MW (0.01 %) and no children. In ambulatory care, only one adult (0.01 %) and no children died. Table 3 presents the mortality trends according to study period and clinical pathways and main characteristics of dead patients.

**Table 3:** Mortality by study period and type of care pathways with main characteristics (type of *Plasmodium* and severity) (Period 1: 2000–2003, Period 2: 2004–2008, Period 3: 2009–2013)

	Period 1	Period 2	Period 3	p value	Period 2 vs Period 1	Period 3 vs Period 1
	n (%)	n (%)	n (%)		Difference (CI 95 %))	Difference (CI 95 %)
Adults^a^	n = 6482	n = 5514	n = 4989			
Overall mortality	24 (0.37)	16 (0.29)	14 (0.28)	0.35	−0.08 % (−0.3; 0.13)	−0.09 % (−0.32; 0.12)
*P. falciparum*	22 (0.34)	16 (0.29)	14 (0.28)	0.50		
Severity–French criteria	19 (0.29)	11 (0.2)	14 (0.28)	0.50		
Severity–clinical classification	21 (0.32)	15 (0.27)	14 (0.28)	0.50		
Medicine ward	2 (0.09)	1 (0.06)	2 (0.15)	0.87		
ICU	14 (8.5)	10 (5.52)	12 (3.96)	0.09		
Ambulatory care	1 (0.05)	0 (0)	0 (0)	0.13		
Children^b^	n = 2066	n = 1308	n = 895			
Overall mortality	2 (0.1)	0 (0)	1 (0.11)	0.52	−0.1 % (−0.47; 0.1)	(0.01 % (−0.5; 0.29)
*P. falciparum*	2 (0.1)	0 (0)	0 (0)	0.33		
Severity–French criteria	0 (0)	0 (0)	1 (0.11)	0.33		
Severity–clinical classification	1 (0.5)	0 (0)	1 (1.1)	0.33		
Medicine ward	0 (0)	0 (0)	0 (0)			
ICU	2 (14.9)	0 (0)	0 (0)	0.006		
Ambulatory care	0 (0)	0 (0)	0 (0)			

Data are number (%)

^a^For five dead adults, the type of pathway was missing. Among hospitalized adults, the type of hospitalization was missing for seven adults

^b^The third dead child was hospitalized but the type of hospitalization was missing

## Discussion

The present study shows a rise in the proportion of severe forms of imported malaria among adults and children, a decrease in hospitalization rate and an increase in ambulatory care among adults, while for both adults and children a rise in ICU admissions. Despite these changes in severity and proposed care pathways, any increase in mortality was found during the study period.

The frequency of severe forms of imported malaria, based on classification by a clinician and according to objective criteria [8, 16], can be determined. According to each of the two definitions of severity, our results indicate increased frequency in severe forms of the disease, both among adults (+6 % and +6 %) and children (+5 % and +6 %). A significant upward trend in the frequency of certain criteria for severity was identified: impaired consciousness, haemodynamic failure, jaundice, increased bilirubin, severe anaemia, hyperlactataemia, renal failure, and parasitaemia >4 %, and a rise in the number of aged cases. All them are recognized as a severity criterion [11, 12, 14–16, 21].

The present study found major changes in the management of patients with imported malaria during the study period. For adults only, a progressive decrease in global hospitalization rates (−7 %) between Period 1 and Period 3, indicating that re-orienting adult subjects from hospitalization towards outpatient care took the form of a switch from MW hospitalizations (−15 %) towards ambulatory care (+7 %). The observed rise in ambulatory care for adults can be interpreted as the consequence of French guidelines which, as early as 1999 [8], suggested ambulatory care for patients exhibiting neither severity criteria nor particular difficulties that could impede outpatient follow-up care. These guidelines were strengthened in 2007 to formally recommend ambulatory care for such patients [16]. However, the biggest effects were observed between Periods 1 and 2, starting in 2000. This suggests that the practice of reducing MW hospitalizations in favour of ambulatory care preceded the 2007 guidelines, which merely confirmed and reinforced pre-existing practices. The availability of new oral anti-malarial treatments could also have played a role. Not significant modification in care pathways for children was observed, either in hospitalization or in ambulatory care. However, the availability of oral anti-malarial treatments adapted to paediatric patients remains limited in France, and the 2007 guidelines excluded paediatric cases [16]. Recently, the WHO has recommended early home-based management with a community health worker for children in endemic zones, thus limiting hospitalization to cases meeting severity criteria [12]. This strategy has shown results in terms of reducing both child mortality and costs [22, 23].

The present study also observed an increase in ICU admissions rates, both for adults (+4 %) and for children (+3 %). For adults, a decrease in global admissions and in MW admissions was associated with an increase in the proportion of ICU admissions. While fewer than one in ten patients had been admitted to an ICU during Period 1, this figure rose to almost one in five patients in Period 3. Similarly, it was noted an increase in ICU admissions for children, but to a lesser extent. The observed rise in severe forms could explain these increasing rates of ICU admission of imported malaria cases. Current guidelines recommend ICU admission for cases presenting at least one of the severity criteria [10–16, 24, 25]. However, these criteria are not clearly distinguished from those indicating hospitalization in a general MW.

The global mortality rate in our study was 0.27 %, that is, 0.32 and 0.007 % for adults and children, respectively, and therefore close to previously reported rates [3–6, 26–28]. ICU mortality (5.47 %) was close to previously reported rates [17, 21, 27, 28]. We found that adult mortality rates in MW and ambulatory care were 0.1 and 0.01 %. No child died in MW or ambulatory care. The mortality rate for patients admitted to MW has not been well documented, while ambulatory care mortality has not previously been reported. These mortalities rates are very low, then ambulatory care can be proposed. Mortality seems to be concentrated among patients admitted directly to ICU, that is, those initially exhibiting the clearest criteria for severity in a clinician’s view. Yet the increased frequency in severe forms and modifications in care pathways, particularly a rise in adult ambulatory care, might have led to fear an increase in mortality rates during the course of the study. On the contrary, no significant change was observed during the study periods in either global mortality or in mortality rates according to clinical pathway, whether among adults or children.

## Conclusions

The results of the present study confirm that care pathways were modified during the course of the study period. For adults, this modification was characterized by a reduction in hospitalizations in general MW and by a rise in ambulatory care; and for adults and children, by an increase in the proportion of patients admitted to an ICU. Most international guidelines call for hospitalization of patients suffering from imported malaria. The present study results underline the importance of better defined criteria for hospitalization in an MW and ICU. The lack of any significant change in mortality indicates that management of imported malaria in Ile-de-France (the global strategy of reducing hospitalizations in MW and developing ambulatory care pathways for adults with non-severe forms), was not associated with a rise in mortality. Then, ambulatory care can be proposed for adults with non-severe imported malaria. The present study provides an answer to an old question concerning the hospitalization of imported malaria [29, 30] and opens up new clinical possibilities and research perspectives in this field.

## Additional files

10.1186/s12936-016-1364-9 French criteria for definition of severe *P. falciparum* malaria case (if at least one of these criteria is met), according to 2007 recommendations.
10.1186/s12936-016-1364-9 Main characteristics and number of cases in the 44 hospitals of the CNR-M in Ile-de-France.
10.1186/s12936-016-1364-9 Flow chart of the study population selection from the French National Reference Center for Malaria database.
10.1186/s12936-016-1364-9 Trends in the number of malaria cases reported in Ile-de-France, 2000–2013, by month, according to age group.
10.1186/s12936-016-1364-9 Trends in proportion of use of new oral treatment (atovaquone–proguanil, artemether–lumefantrine, dihydroartemisinin–piperaquine) in first intention in adult cases, by study periods and type of care pathway.
10.1186/s12936-016-1364-9 Trends in the proportion of severe cases (according to French criteria) among malaria cases reported in Ile-de-France, 2000–2013, by month, according to age group. The number reported above the curve is the observed proportion for each period (Period 1: 2000–2003, Period 2: 2004–2008, Period 3: 2009–2013).
