# Supplementary material for: Hospitalization and ambulatory care in imported-malaria: evaluation of trends and impact on mortality. A prospective multicentric 14-year observational study
Source: Malar J. 2016 Jun 7;15:312. doi: 10.1186/s12936-016-1364-9 (PMC4897798; doi:10.1186/s12936-016-1364-9)
Supplement: Supplementary file 1 — 10.1186/s12936-016-1364-9 French criteria for definition of severe P. falciparum malaria case (if at least one of these criteria is met), according to 2007 recommendations. [file 12936_2016_1364_MOESM1_ESM.docx]

**Additional file 1**: **French criteria for definition of severe *P. falciparum* malaria case (if at least one of these criteria is met), according to 2007 recommendations**

| **Clinical features** | **Prognostic value** | **Frequency** | **Corresponding variable in the CRF** |
| --- | --- | --- | --- |
| Cerebral malaria  - confusion, drowsiness, prostration  - Glasgow coma scale <11 | **+++** | **+++** | Impaired consciousness (confusion, drowsiness, prostration)  Glasgow coma scale <11 |
| Respiratory failure  - PaO2/FiO2 <300 mmHg (under mechanical ventilation)  - PaO2 <60 mmHg and/or SpO2 <90% (ambient air) and / or respiratory frequency >32/min  - radiological signs | **+++** | **+** | Respiratory failure (acute respiratory distress syndrome, pulmonary oedema) |
| Cardiovascular failure  - systolic blood pressure <60 mmHg in children <5 years old; <80 mmHg in adults with signs of peripheral circulatory deficiency  - vasoactive treatment  - peripheral circulatory deficiency without low blood pressure | **+++** | **++** | Cardio circulatory shock |
| Repeated generalized seizures  ≥2 seizures/24 hours | **++** | **+** | Repeated generalized seizures: ≥2 seizures/24 hours |
| Haemorrhage | **++** | **+** | Haemorrhage (abnormal bleeding, including CIVD) |
| Jaundice | **+** | **+++** | Jaundice |
| Macroscopic haemoglobinuria | **+** | **+** | Macroscopic haemoglobinuria |
| **Laboratory findings** |  |  |  |
| Severe normocytic anaemia: haemoglobin <7 g/dL  with haematocrit <20% | **+** | **+** | Haemoglobin <7 g/dL, haematocrit <20% |
| Hypoglycaemia  blood glucose <2.2 mmol/L (<40 mg/dL) | **+** | **+** | Blood glucose <2.2 mmol/L or severe hypoglycaemia according to physician’s judgment |
| Acidaemia: pH <7.35 or acidosis: plasma bicarbonate <15 mmol/L | **+++** | **++** | Acidaemia: pH <7.35 |
| Hyperlactataemia: lactate >5 mmol/L | **+++** | **++** | All hyperlactataemia, a fortiori if lactates >5 mmol/L |
| Acute renal failure  - urine output <400 mL/24 hours in adults (12 mL/kg/24 hours in children), not improved despite rehydration  - and with serum creatinine >265 µmol/L | **++** | **+++** | Creatinaemia >265 µmol/L or severe acute renal failure according to physician’s judgment |
| Hyperparasitaemia: >4% | **+** | **+++** | Parasitaemia >4% |

Prognostic and frequency are those indicated in 2007 French recommendations and are the results of a French cohort of patients admitted in ICU between 1988 and 1999.^3^
