# Supplementary material for: Hospitalization and ambulatory care in imported-malaria: evaluation of trends and impact on mortality. A prospective multicentric 14-year observational study
Source: Malar J. 2016 Jun 7;15:312. doi: 10.1186/s12936-016-1364-9 (PMC4897798; doi:10.1186/s12936-016-1364-9)
Supplement: Supplementary file 2 — 10.1186/s12936-016-1364-9 Main characteristics and number of cases in the 44 hospitals of the CNR-M in Ile-de-France. [file 12936_2016_1364_MOESM2_ESM.docx]

**Additional file 2: Main characteristics and number of cases in the 44 hospitals of the CNR-M in Ile-de-France**

| Health Care Facility | Academic hospitals | Number of imported cases notified (2000-2013) | Mean number of cases/year |
| --- | --- | --- | --- |
| Aulnay-sous-Bois |  | 1034 | 74* |
| Beaujon | X | 9 | 9 |
| Bicêtre | X | 1138 | 81* |
| Bobigny-Avicenne | X | 720 | 51* |
| Bondy-Jean Verdier | X | 809 | 58* |
| Boulogne | X | 375 | 27* |
| Bry-sur-Marne |  | 306 | 61 |
| Clamart | X | 309 | 22* |
| CMTE |  | 22 | 3 |
| Colombes-Louis Mourier | X | 674 | 48* |
| Corbeil Essonne |  | 280 | 20* |
| Creteil-CHIC |  | 397 | 57 |
| Creteil-Henri Mondor | X | 467 | 33* |
| Dourdan |  | 32 | 2 |
| Evry |  | 172 | 29 |
| Fontainebleau |  | 86 | 6* |
| Gonesse |  | 293 | 73 |
| Lagny-sur-Marne |  | 142 | 24 |
| Longjumeau |  | 263 | 22 |
| Mantes-la-Jolie |  | 92 | 13 |
| Meaux |  | 209 | 15* |
| Meulan-Les Mureaux |  | 77 | 15 |
| Nanterre |  | 28 | 9 |
| Paris-Bichat | X | 2628 | 188* |
| Paris-Cochin | X | 508 | 36* |
| Paris-HEGP | X | 266 | 19* |
| Paris-HIA Begin |  | 869 | 62* |
| Paris-Hôtel-Dieu | X | 46 | 9* |
| Paris-Institut Pasteur |  | 224 | 16* |
| Paris-Lariboisière | X | 290 | 58* |
| Paris-Necker | X | 503 | 36* |
| Paris-Percy |  | 1 | 1 |
| Paris-Pitié-Salpêtrière | X | 1892 | 135* |
| Paris-Robert Debré | X | 1027 | 73* |
| Paris-Saint-Antoine | X | 536 | 38* |
| Paris-Saint-Louis | X | 600 | 43* |
| Paris-Tenon | X | 819 | 59* |
| Paris-Trousseau | X | 472 | 34* |
| Paris-Saint Joseph |  | 48 | 24 |
| Pontoise |  | 49 | 12 |
| Provins |  | 24 | 3 |
| Saint-Denis |  | 1494 | 107* |
| Versailles |  | 426 | 30* |
| Villeneuve Saint-Georges |  | 730 | 52* |
| TOTAL |  | 21 386 |  |

* Hospitals which reported malaria cases each year during the 14-year study period.
