# Supplementary material for: Hospitalization and ambulatory care in imported-malaria: evaluation of trends and impact on mortality. A prospective multicentric 14-year observational study
Source: Malar J. 2016 Jun 7;15:312. doi: 10.1186/s12936-016-1364-9 (PMC4897798; doi:10.1186/s12936-016-1364-9)
Supplement: Supplementary file 3 — 10.1186/s12936-016-1364-9 Flow chart of the study population selection from the French National Reference Center for Malaria database. [file 12936_2016_1364_MOESM3_ESM.docx]

**Additional file 3: Flow chart of the study population selection from the French National Reference Center for Malaria database**

CNR Malaria Ile-de-France 2000-2013

**n= 21,386**

**Adults: 16,985 ; Children: 4,269**

**Severity**

Medical classification

Missing values 781

n= 1,031

Adults 871 ; Children 159

Ambulatory

Missing values 2,374

Admitted to MW

Missing values 2,963

**Care pathway**

**Mortality**

n= 2172

Adults 1,563 ; Children 602

French criterion

Missing values 0

n= 7,804

Adults 6,952 ; Children 817

n= 7503

Adults 5,126 ; Children 2,338

Admitted to ICU

Missing values 2,963

n= 742

Adults 658 ; Children 84

Dead

Missing values 0

n=57

Adults 54 ; Children 3
