# Supplementary material for: Hospitalization and ambulatory care in imported-malaria: evaluation of trends and impact on mortality. A prospective multicentric 14-year observational study
Source: Malar J. 2016 Jun 7;15:312. doi: 10.1186/s12936-016-1364-9 (PMC4897798; doi:10.1186/s12936-016-1364-9)
Supplement: Supplementary file 4 — 10.1186/s12936-016-1364-9 Trends in the number of malaria cases reported in Ile-de-France, 2000–2013, by month, according to age group. [file 12936_2016_1364_MOESM4_ESM.docx]

**Additional file 4: Trends in the number of malaria cases reported in Ile-de-France, 2000-2013, by month, according to age group.**


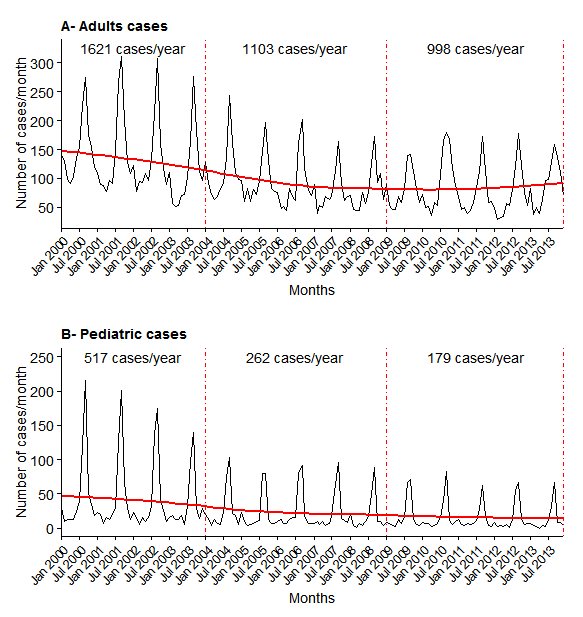
The figure shows the number of malaria cases reported each month to the CNR between January 1^st^, 2000 and December 31, 2013 (black lines), smoothed with a 2 degree polynomial regression line (red lines), in adults (A) and pediatric cases (B). Dotted red lines represent limits between the three study periods. Average numbers of cases per year are specified for each period.
