# Supplementary material for: Hospitalization and ambulatory care in imported-malaria: evaluation of trends and impact on mortality. A prospective multicentric 14-year observational study
Source: Malar J. 2016 Jun 7;15:312. doi: 10.1186/s12936-016-1364-9 (PMC4897798; doi:10.1186/s12936-016-1364-9)
Supplement: Supplementary file 5 — 10.1186/s12936-016-1364-9 Trends in proportion of use of new oral treatment (atovaquone–proguanil, artemether–lumefantrine, dihydroartemisinin–piperaquine) in first intention in adult cases, by study periods and type of care pathway. [file 12936_2016_1364_MOESM5_ESM.docx]

**Additional file 5: Trends in proportion of use of new oral treatment (atovaquone-proguanil, artemether-lumefantrine, dihydroartemisinin-piperaquine) in first intention in adult cases, by study periods and type of care pathway**


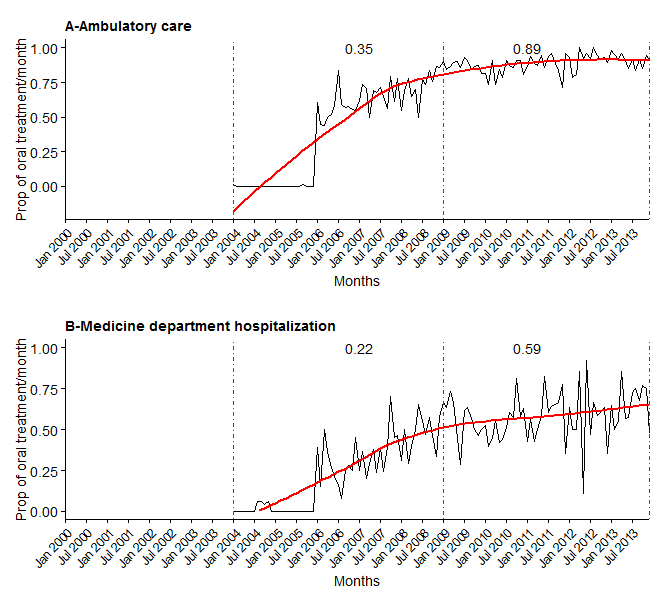
The figure shows the proportion of patients who received oral treatment (atovaquone-proguanil, artemether-lumefantrine, dihydroartemisinin-piperaquine) in first intention among adults cases in ambulatory care (A) or hospitalized in a general Medical Ward, by study period (black lines), smoothed with a 2 degree polynomial regression line (red lines). Dotted red lines represent limits between the three study periods. Proportions are those observed during each study period.
