# Supplementary material for: Hospitalization and ambulatory care in imported-malaria: evaluation of trends and impact on mortality. A prospective multicentric 14-year observational study
Source: Malar J. 2016 Jun 7;15:312. doi: 10.1186/s12936-016-1364-9 (PMC4897798; doi:10.1186/s12936-016-1364-9)
Supplement: Supplementary file 6 — 10.1186/s12936-016-1364-9 Trends in the proportion of severe cases (according to French criteria) among malaria cases reported in Ile-de-France, 2000–2013, by month, according to age group. The number reported above the curve is the observed proportion for each period (Period 1: 2000–2003, Period 2: 2004–2008, Period 3: 2009–2013). [file 12936_2016_1364_MOESM6_ESM.docx]

**Additional file 6 : Trends in the proportion of severe cases (according to French criteria) among malaria cases reported in Ile-de-France, 2000-20013, by month, according to age group. The number reported above the curve is the observed proportion for each period (Period 1: 2000-2003, Period 2: 2004-2008, Period 3: 2009-2013)**

**
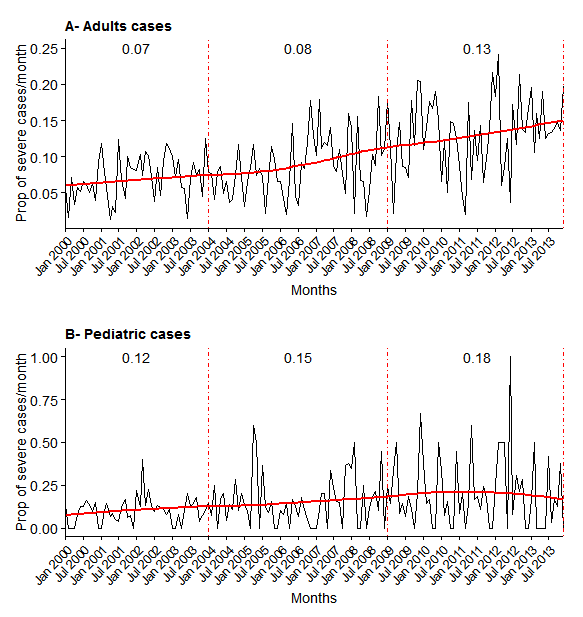
**

The figure shows the proportion of severe malaria cases by month among cases reported to the CNR between January 1, 2000 and December 31, 2013 (black lines), smoothed with a 2 degree polynomial regression line (red lines), in adults (A) and pediatric cases (B). Dotted red lines represent limits between the three study periods.
